# The Cedar Project: exploring determinants of psychological distress among young Indigenous people who use drugs in three Canadian cities

**DOI:** 10.1017/gmh.2018.26

**Published:** 2018-10-30

**Authors:** M. E. Pearce, K. A. Jongbloed, S. D. Pooyak, A. H. Blair, W. M. Christian, R. Sharma, A. Mazzuca, D. S. Zamar, M. T. Schechter, P. M. Spittal, for the Cedar Project Partnership

**Affiliations:** 1 Canadian HIV Trials Network, Vancouver, BC, Canada; 2 BC Children's Hospital Research Institute, Vancouver, BC, Canada; 3 University of British Columbia School of Population and Public Health, Vancouver, BC, Canada; 4 Cree, University of Victoria, Canadian Aboriginal AIDS Network, Victoria, BC, Canada; 5 Splatsin te Secwepemc First Nation, Enderby, BC, Canada

**Keywords:** Childhood maltreatment, drug use, Indigenous young people, psychological distress

## Abstract

**Background.:**

Mental health and wellbeing, including addressing impacts of historical trauma and substance use among young people, has been identified as a key priority by Indigenous communities and leaders across Canada and globally. Yet, research to understand mental health among young Indigenous people who have used drugs is limited.

**Aims.:**

To examine longitudinal risk and strengths-based factors associated with psychological distress among young Indigenous people who use drugs.

**Method.:**

The Cedar Project is an ongoing cohort study involving young Indigenous people who use drugs in Vancouver, Prince George, and Chase, British Columbia, Canada. This study included participants who completed the Symptom Checklist-90-Revised, returned for follow-up between 2010 and 2012, and completed the Childhood Trauma Questionnaire. Adjusted linear mixed-effects models estimated effects of study variables on changes in area T-scores of psychological distress.

**Results.:**

Of 202 eligible participants, 53% were women and the mean age was 28 years. Among men, childhood maltreatment (emotional abuse, physical abuse, sexual abuse, physical neglect), any drug use, blackouts from drinking, and sex work were associated with increased distress. Among women, childhood maltreatment (emotional abuse, physical abuse, physical neglect), blackouts from drinking, and sexual assault were associated with increased distress, while having attempted to quit using drugs was associated with reduced distress. Marginal associations were observed between speaking their traditional language and living by traditional culture with lower distress among men.

**Conclusion.:**

Culturally safe mental wellness interventions are urgently needed to address childhood trauma and harmful coping strategies that exacerbate distress among young Indigenous people who use drugs.

## Introduction

Mental health and wellbeing, including addressing the impacts of historical trauma and substance use among young people, has been identified as a key priority by Indigenous communities and leaders across Canada and globally (New Zealand Ministry of Health, 2008; Purdie *et al*., 2010; Assembly of First Nations, 2015). Indigenous peoples in British Columbia (BC), Canada, have enduring traditions of health rooted in perspectives of wellness that incorporate emotional, physical, mental, and spiritual realms, and extend beyond the individual to include both family and community (Henderson, 2008; First Nations Health Authority, 2013). Stress-coping strategies passed down through generations of Elders to young people supported connections to land, community, culture, and spirituality (Gunn Allen, 1986; Chansonneuve, 2005). However, mental wellness was disrupted by colonization and its aggressive legal, ideological and economic agendas. These were meant to impede and delegitimize Indigenous political, social, and cultural systems; dispossess Indigenous communities from lands and resources; and assimilate Indigenous peoples into settler culture (Kelm, 1998). Indigenous scholars have characterized the impact of colonization on Indigenous peoples as historical and intergenerational trauma, defined as a collective emotional and psychological injury over the lifespan and across generations (Yellow Horse Brave Heart, 2003). A powerful mechanism of colonization in Canada was the residential school system, implemented from 1874 to 1996, which has had lasting impacts on mental wellness among Indigenous peoples. Over 150 000 Indigenous children were forcibly removed from their communities and placed in residential schools as part of a national strategy to assimilate without interference by their parents, Elders, and leaders (Miller, 2009). Children in the schools routinely faced predation by missionary teachers and staff (Milloy, 1999; Hylton, 2002) and were taught to feel shame about their cultures, languages, and identities (Truth and Reconciliation Commission Canada, 2015*b*). Psychological effects of having been in the residential school system include major depression (Public Health Agency of Canada, 2006; Froese *et al*., 2008), post-traumatic stress disorder (Corrado & Cohen, 2003; Söchting *et al*., 2007), problematic substance use (Ross *et al*., 2015), and suicidality (Royal Commission on Aboriginal Peoples, 1995). Wounds have often been intergenerational, as many residential school survivors inadvertently re-enacted painful experiences in their own families (Truth and Reconciliation Commission Canada, 2015*a*). Ongoing manifestations of colonization have meant that few resources exist to support the children and grandchildren of survivors. Disproportionate numbers of Indigenous children in Canada experiencing the traumas of abuse and neglect is considered one of the most disastrous legacies of the system (For the Cedar Project Partnership *et al*., 2008; Trocmé & Wolfe, 2010). Moreover, Indigenous scholars have long argued that the residential school system has been replaced by provincial child welfare systems, which has been associated with negative health outcomes (Fournier & Crey, 1997; Blackstock, 2007; For the Cedar Project Partnership *et al*., 2015). Though only 10% of children in BC are Indigenous, they comprised 60% of children in foster care in 2016 (Grand Chief Ed John, 2016).

Psychological literature has hypothesized that pathways by which lifetime trauma leads to problematic substance use and psychological distress involve insecure attachment to family and community; internalization of blame and powerlessness; harmful coping strategies; and reluctance to seek social support (Barker-Collo and Read, 2003; Cohen *et al*., 2003). Epidemiological literature has subsequently explored the relationship between psychological distress and heightened vulnerability to HIV and Hepatitis C (HCV) infection within high risk drug use environments. This research highlights relationships between trauma and addiction (Dube *et al*., 2003), and addiction and mental illness (Medrano *et al*., 2002). Researchers have focused on the effect that concurrent addiction and psychological distress have on unsafe sex or risky drug use practices, and subsequent vulnerability to HIV and HCV infection (Meade *et al*., 2009; Pilowsky *et al*., 2011). For example, a cross-sectional study involving 155 American Indian women in metropolitan New York demonstrated that injection drug use mediated the relationship between sexual trauma and sexual behaviours related to HIV infection (Simoni *et al*., 2004).

Despite the complex intersections of colonial and lifetime trauma, substance use, and psychological distress, many Indigenous cultures, traditions, and languages have survived and promote mental wellness among individuals, families, and communities. For example, Snowshoe *et al*. (2017) identified that factors of cultural connectedness including Indigenous identity, traditions, and spirituality were strongly associated with greater self-efficacy, sense of self, school connectedness, and life satisfaction among 290 Indigenous youth in Saskatchewan and Ontario, Canada. Further, programs that include ‘culture as healing’ and build on strengths-based approaches to stress-coping founded in Indigenous practices have made a positive impact (Marsh *et al*., 2015; McKenzie *et al*., 2016). Yet, little is known about the negative or positive influence that social determinants of health have on the mental well-being of young, urban Indigenous people who use drugs. There are significant gaps in mental health research involving Indigenous people in Canada, especially those living off-reserve (Bombay, 2015; Nelson & Wilson, 2017). Currie *et al*. (2013*a*) demonstrated that within a national sample of young Indigenous people living off-reserve in Canada, participants were more likely to use prescription drugs to get high compared with non-Indigenous youth and were less likely to feel connected to their school. To our knowledge, no studies have explored both strengths-based and risk factors associated with psychological distress among young Indigenous people who have been impacted by substance use. The present study examined the longitudinal effects of historical and lifetime traumas, strengths-based factors (including Indigenous cultures, traditions, and languages), and drug- and sex-related vulnerabilities on psychological distress among young Indigenous people in urban Canada.

## Methods

Cedar Project methods have been described in detail elsewhere (Spittal *et al*., 2007). In brief, the Cedar Project is a cohort involving young Indigenous people who use illicit drugs in Vancouver, Prince George, and Chase, BC. Vancouver is a large city in southern BC, on the traditional territory of the Coast Salish peoples. Prince George is a mid-sized city in the northern interior of BC, on the traditional territory of Lheidli T'enneh First Nation. Chase is a rural town in south-western BC, on the traditional territory of Secwepemc Nation. Participants were recruited with the help of health care providers as well as by street outreach and word of mouth. Participants were eligible if they self-identified as a descendant of Indigenous Peoples of North America; were 14–30 years old; had smoked/injected drugs in the month before enrolment; and provided written informed consent. Since 2003, participants have returned every 6 months to complete interviewer-administered questionnaires and provide venous blood samples, which are tested for HIV and HCV. Honoraria are provided at each follow-up visit. Indigenous collaborators and investigators, collectively known as the Cedar Project Partnership, governed the entire research process and approved this manuscript for publication. The University of British Columbia/Providence Health Care Research Ethics Board also approved the study.

## Measures

This longitudinal analysis includes time-invariant measures collected when Cedar Project participants entered the study (e.g. sex, city of enrollment) and time-variant measures (e.g. recent injection drug use) collected every 6 months thereafter. Psychometric questionnaires were introduced as part of regular 6-month follow-up visits for all participants in 2010. As a result, this analysis here involves time-varying data collected during three follow-up visits between 2010 and 2012.

### Psychological distress

The Symptom Checklist-90-R (SCL-90-R) (Derogatis, 1994) was added to the Cedar Project package of questionnaires in 2010, and has been administered at every 6-month follow-up visit since. The SCL-90-R is a 90-item self-reported symptom inventory that measures severity of nine dimensions of psychological distress symptoms including somatization, obsessive–compulsive, interpersonal sensitivity, depression, anxiety, hostility, phobic anxiety, paranoid ideation, and psychoticism. Each item is scored on a five-point Likert scale that quantified the degree of distress caused by symptoms in the past 3 months (from *not at all* to *extremely*). The sum of scores was divided by 90 to determine an average Global Severity Index (GSI) score which reflects the number of distress symptoms an individual is experiencing as well as the intensity of distress. The SCL-90-R manual provides tables to transform the subscales and the GSI to standardized area T-scores based on normative samples (Derogatis, 1994). The current study used normative data from the psychiatric outpatient sample (425 males, 577 females, 32.9% non-white, 63.4% with lower socioeconomic status) of adults presenting to mental health outpatient clinics with symptoms not requiring hospitalization, as the distribution of outpatient scores were most comparable to our sample. Raw GSI score means were plotted according to the transformed standardized area T-score to allow for comparison of Cedar Project participants to adult psychiatric outpatients. The area T-scores can then be used to interpret the percentile position of participants relative to the norm population. For example, an area T-score of 60 will always place individuals in the 84th centile of the normative sample and a T-score of 70 will place them in the 98th percentile.

The SCL-90- has been used to successfully estimate psychological distress symptoms among Indigenous young people globally (Howard-Pitney *et al*., 1992; Yen *et al*., 2006). In addition, the SCL-90-R was validated for use among Cedar Project participants (Pearce, 2014). The internal reliability of coefficients for the nine clinical scales of the SCL-90-R symptom dimensions were very good: *α* =  0.915 for somatization, *α* =  0.906 for obsessive–compulsive, *α* =  0.906 for interpersonal sensitivity, *α* =  0.929 for depression, *α* =  0.924 for anxiety, *α* =  0.878 for hostility, *α* =  0.872 for phobic anxiety, *α* =  0.856 for paranoid ideation, and *α* =  0.895 for psychoticism.

### Historical and intergenerational trauma

We used two time-invariant proxy measures of historical and intergenerational trauma: having at least one parent who attended residential school (no *v*. unsure *v*. yes) and ever having been taken away from biological parents and placed in foster care (no *v*. yes) (For the Cedar Project Partnership *et al*., 2008).

### Childhood maltreatment

Since 2011, participants have been offered the option of completing the Childhood Trauma Questionnaire (CTQ) (Bernstein & Fink, 1998). The CTQ is a self-reported 28-item inventory measuring five types of childhood maltreatment: emotional abuse, physical abuse, sexual abuse, emotional neglect, and physical neglect. Responses are provided using a five-point Likert scale according to frequency of experiences (*never true* to *very often true*). Participants completed the CTQ only once. In the current study, due to floor and ceiling effects within subscales, the maltreatment scores were converted into three levels of maltreatment – none, low/moderate, and severe. The CTQ has been validated among adults who use drugs (Thombs *et al*., 2007), and homeless young people in Canada (of whom 12% identified as Indigenous people) (Forde *et al*., 2012). In addition, the CTQ has been validated among Cedar Project Participants (Pearce, 2014). For this study sample, the internal reliability of the coefficients for the five clinical scales of the CTQ maltreatment types were acceptable: *α* = 0.880 for emotional abuse, *α* = 0.900 for physical abuse, *α* = 0.962 for sexual abuse, *α* = 0.850 for emotional neglect, and *α* = 0.702 for physical neglect.

## Other study variables

Independent study variables were chosen based on theoretical and empirical importance. Fixed variables included biological sex (male *v*. female); study location (Prince George *v*. Chase *v*. Vancouver); education level (less than high school *v*. high school graduate or more); frequency family had lived by traditional culture (never/rarely *v*. often/always); frequency family had spoken traditional languages at home (never/rarely *v*. often/always); and ability to speak own traditional language (no *v*. a little bit *v*. yes). Living by traditional culture was defined as living according to values inherent to customary Indigenous ways of life and taught by Elders, including humility, honesty, love, respect, loyalty, remembering where you are from, and putting family first. These variables were defined by two Indigenous Elders, Earl Henderson (Cree/Métis) and Violet Bozoki (Lheidli T'enneh First Nation), who are traditional Knowledge Keepers, and members of the Cedar Project Partnership.

Non-fixed, or longitudinal variables related to the previous 6-month period (‘recent’) and included age; relationship status (single *v*. in a relationship); frequency of living by traditional culture (never/rarely *v*. often/always); participating in traditional ceremonies (never/rarely *v*. often/always); accessing alcohol/drug treatment (no *v*. yes); accessing counseling (no *v*. yes); trying to quit using drugs (no *v*. yes); sleeping on the streets for three or more consecutive nights (no *v*. yes); frequency of crack smoking (less than daily *v*. daily or more); any injection drug use (no *v*. yes); binge drinking (no *v*. yes); blackouts from drinking (no *v*. yes); sex work involvement (no *v*. yes); consistent condom use with regular or casual partners (always *v*. not always); having a sexually transmitted infection (no *v*. yes); having been sexually assaulted (no *v*. yes); and HIV and HCV serostatus. Participating in traditional ceremonies included potlatch, feast, fast, burning ceremony, sweat lodge, washing ceremony, naming ceremony, big/smoke house, rites of passage, smudge, dances, or any other traditional Indigenous ceremony. Binge drinking was defined as having gone on runs or binges of drinking more than usual. Regular partners were defined as those with whom participants had sexual relationships lasting 3 months or more, and casual partners were those lasting <3 months. Sexually transmitted infections were self-reported and may have included chlamydia, genital warts, gonorrhea, herpes, syphilis, or others.

## Statistical analysis

Frequency distributions described demographic and historical and intergenerational trauma factors for all participants and for male and female participants separately. The χ^2^ tests identified significant differences between males and females. Raw symptom dimension distress and GSI scores were calculated according to the above-mentioned formulae. Welch's *t* test was used to test for differences in raw mean psychological distress scores between males and females.

Linear mixed-effects modeling was used to examine change over time in mean psychological distress score (area T-score for the GSI) using the lme4 package (Bates, Maechler, Bolker, & Walker, 2015) in R Version 3.3.3 (R Core Team, 2016). Sex-stratified unadjusted and adjusted LME models estimated the independent effect of each study variable on mean change in psychological distress over the study period between 2010 and 2012. Thus, separate models were fit for each study variable of interest. This strategy for model building, called purposeful selection, was used to avoid the problem of overfitting (Bursac *et al*., 2008). Finally, sex-stratified joint models were obtained via likelihood ratio test (LRT), wherein all variables that were significant at the 0.10 level after adjustment were considered for inclusion. Nested models were compared via analysis of variance (ANOVA). Variables not found to significantly improve the models were sequentially dropped one at a time.

In all models, participants were specified as a random factor (i.e. random intercept models) to control for their associated intraclass correlation (Pinheiro & Bates, 2000). Associations between variables and psychological distress were tested in both unadjusted and adjusted analyses after controlling for confounding. Potential confounders that were tested included: sexual identity, parental attendance at residential school, ever been in foster care, location, relationship status, and education level. Confounders were selected if their inclusion produced a change of 15% or greater in the estimated effect of the exposure of interest. Confounders identified include age, location, and education level, which were included in each multivariable model. The *p* values were calculated using the LRT. As this study is largely hypothesis-driven, we chose not to correct for multiple testing. Naturally, additional dedicated studies are needed to confirm the results.

### Missing data

A total of 10 observations (1.2%) with more than 20% missing data in the SCL-90-R were removed (Derogatis, 1994). SCL-90-R questionnaires with data missing at random may be validly scored if the actual number of responses (rather than total number) are summed for the denominator in the division of summed scores. The remaining study variables had missing data ranging from 0.05% to 3.7% of observations.

## Results

In total, 811 participants enrolled in the Cedar Project from 2003 to 2012. This study focused on the time period between 2010 and 2012, during which 423 participants returned for at least one follow-up interview and therefore completed a baseline SCL-90-R questionnaire. Among those participants, 246 (58%) returned for at least two follow-up interviews and were retained in this study. Additional criteria of having completed the CTQ reduced the sample to 212. Finally, 10 observations had >20% missing data in the SCL-90-R and were removed. Therefore, the final sample included 202 participants (48%).

Baseline characteristics of participants stratified by sex are presented in Table 1. Mean age in 2010 was 28.5 years (s.d 5.0) and 53% were women. Nearly half (49%) had at least one parent who attended residential school and most participants (70%) had been in the foster care system. The majority had less than high school education (81%) and were in a relationship (91%). No differences in baseline characteristics were observed between men and women.

**Table 1.** Comparison of frequencies for potential confounders between males (n  =  95) and females (n  =  107).

	All participants		Male participants		Female participants		
Potential confounder	*n*	%	*n*	%	*n*	%	*p*
Mean age (s.d)	28.52	5.02	28.73	5.39	28.34	4.68	0.079
Sexual identity							
Straight	178	88	84	88	94	88	1.00
LGBTQ	24	12	11	12	13	12	
At least one parent attended residential school							
No	43	21	20	21	23	22	0.801
Unsure	59	29	30	32	29	27	
Yes	99	49	45	47	54	51	
Ever in foster care							
No	61	30	30	32	31	29	0.687
Yes	141	70	65	68	76	76	
Location							
Prince George/Chase	127	63	55	58	72	67	0.190
Vancouver	75	37	40	42	35	33	
Education level							
Less than high school	169	85	76	80	93	89	0.094
High school education or higher	31	16	19	20	12	11	
Relationship status							
Single	19	10	11	12	8	8	0.340
In relationship	181	91	84	88	97	92	

With respect to childhood maltreatment (Table 2), 70% of participants had been emotionally abused, among whom 35% had severe emotional abuse; 58% of participants had been physically abused, among whom 42% had severe physical abuse; 58% had been sexually abused, among whom 40% had severe sexual abuse; 72% had been emotionally neglected, among whom 19% had severe emotional neglect; and 78% had been physically neglected, among whom 38% had severe physical neglect. Greater proportions of women than men had had severe experiences of each type of maltreatment.

**Table 2.** Comparison of baseline frequencies for types of childhood maltreatment between males (n  =  95) and females (n  =  107).

	All participants		Male participants		Female participants		
Childhood maltreatment types and severity levels	*n*	%	*n*	%	*n*	%	*P*
Emotional abuse							
None	57	29	40	43	17	17	
Low/moderate	69	35	35	37	34	34	<0.001
Severe	69	35	19	20	50	50	
Physical abuse							
None	83	42	49	52	34	33	
Low/moderate	32	16	13	14	19	18	0.025
Severe	82	42	32	34	50	49	
Sexual abuse							
None	82	42	57	61	25	25	
Low/moderate	35	18	17	18	18	18	<0.001
Severe	77	40	20	21	57	57	
Emotional neglect							
None	55	28	28	30	27	26	
Low/moderate	103	53	48	52	55	53	0.701
Severe	38	19	16	17	22	21	
Physical neglect							
None	42	22	26	28	16	15	
Low/moderate	78	40	39	43	39	37	0.008
Severe	75	38	25	28	50	48	

**Fig. 1.**
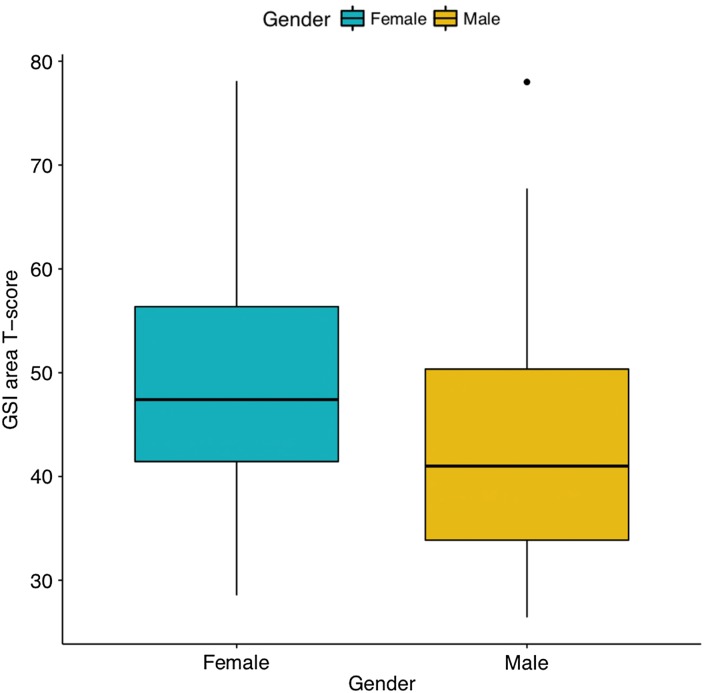
Boxplot of area T-score for the Global Severity Index of psychological distress among Cedar Project participants.

Table 3 presents the sex-stratified ranges, mean raw scores, and standard deviations (s.d) for each of the SCL-90-R symptom dimension scores and the GSI of psychological distress, as well as corresponding area T-scores for the outpatient norm samples. Women had higher scores than men for every symptom dimension. Men scored highest for obsessive–compulsive and hostility, while women scored highest for depression. Figure 1 displays the boxplot for the distribution of the GSI area T-scores for male and female Cedar Project participants. When compared with outpatient norm samples, men in the Cedar Project had a median GSI area T-score of 45 (30th percentile) and women in the Cedar Project had a median GSI area T-score of 48 (42nd percentile). This suggests that men in the Cedar Project were less distressed than the outpatient norm sample, but still comparable. Women in the Cedar Project experienced comparable levels of distress to the outpatient norm sample.

**Table 3.** Mean raw psychological distress subscale scores for Cedar Project participants and transformed standardized area T-scores according to psychiatric outpatient norms (Derogatis, 1994).

	Male participants (*n* = 95)			Female participants (*n* = 107)		
Psychological distress symptom dimensions	Raw scores		T-scores Outpatient norms	Raw scores		T-scores Outpatient norms
	Range	Mean (s.d.)		Range	Mean (s.d)	
Somatization	0–3.42	0.79 (0.75)	54	0–3.75	1.18 (0.93)	54
Obsessive–compulsive	0–3.7	0.92 (0.85)	45	0–4.0	1.3 (1.0)	49
Interpersonal sensitivity	0–4.0	0.75 (0.87)	45	0–4.0	1.15 (0.98)	48
Depression	0–3.62	0.84 (0.83)	43	0–3.85	1.35 (1.01)	45
Anxiety	0–4.0	0.71 (0.85)	44	0–3.9	1.07 (0.99)	51
Hostility	0–4.0	0.87 (0.85)	52	0–4.0	1.28 (1.08)	53
Phobic anxiety	0–3.43	0.57 (0.82)	52	0–4.0	0.84 (0.89)	53
Paranoid ideation	0–3.83	0.85 (0.93)	49	0–4.0	1.11 (1.03)	50
Psychoticism	0–3.6	0.58 (0.81)	47	0–3.7	0.83 (0.88)	51
Global Severity Index	0.01–3.5	0.79 (0.76)	45	0.01–3.71	1.15 (0.88)	48

### Factors associated with psychological distress among men

Table 4 presents sex-stratified unadjusted and adjusted LME models. All types of childhood maltreatment except emotional neglect were strongly associated with elevated mean psychological distress scores among men. Associations were observed between both low/moderate (*β* = 10.44) and severe (*β* =  9.69) emotional abuse and mean increased distress. This translated to a 2.33-fold increase in psychological distress (percentile shift: 30th to 70th). Men who reported severe childhood physical abuse experienced a 1.93-fold increase in distress (*β* = 7.73; percentile shift: 30th to 58th). Those who reported childhood sexual abuse (*β* = 6.12) or severe physical neglect (*β* = 6.74) experienced 1.8-fold increases in distress (percentile shift: 30th to 54th).

**Table 4.** Stratified unadjusted and adjusted^a^ coefficient estimates for each study variable on psychological distress scores among male (n  =  95) and female participants (n  =  107).

	Male participants (*n* = 95)						Female participants (*n* = 107)					
	*β*	95% CI^b^	*p*	Adj. *β*	95% CI^b^	*p*	*β*	95% CI^c^	*p*	Adj. *β*	95% CI^c^	*p*
*Potential confounders*												
Mean age (s.d)	−0.17	−0.58 to 0.25	0.431				−0.27	−0.73 to 0.19	0.251			
LGBTQ sexual identity	0.49	−7.04 to 8.01	0.900				1.79	−4.97 to 8.55	0.605			
Parents attended residential school												
Unsure	1.76	−4.72 to 8.23	0.599				−5.78	−12.08 to 0.52	0.076			
At least one parent attended	1.00	−5.03 to 7.03	0.748				−3.69	−9.31 to 1.93	0.204			
Ever in foster care	−2.68	−7.61 to 2.25	0.290				0.09	−4.79 to 4.96	0.972			
Location (Vancouver)	2.61	−2.02 to 7.24	0.273				−2.85	−7.53 to 1.83	0.235			
HS education or higher	3.70	−2.00 to 9.41	0.207				−3.23	−10.26 to 3.79	0.369			
In a relationship	−2.31	−7.20 to 2.56	0.352				−5.01	−10.69 to 0.67	0.085			
*Childhood maltreatment*												
Emotional abuse severity												
Low/moderate	9.83	5.07–14.59	<0.001	10.44	5.87–15.02	<0.001	4.38	−2.05 to 10.81	0.187	4.14	−2.28 to 10.57	0.219
Severe	8.70	2.98–14.40	0.004	9.69	4.07–15.32	0.001	10.32	4.25–16.39	0.001	10.78	4.70–16.87	0.001
Physical abuse severity												
Low/moderate	1.35	−5.33 to 8.03	0.694	3.89	−2.93 to 10.71	0.277	2.40	−4.03 to 8.83	0.469	2.84	−3.91 to 9.58	0.421
Severe	7.34	2.46–12.22	0.004	7.73	2.97–12.49	0.003	5.48	0.50–10.46	0.034	5.88	0.87–10.88	0.026
Sexual abuse severity												
Low/moderate	3.74	−2.34 to 9.82	0.234	3.44	−2.72 to 9.61	0.287	0.63	−6.40 to 7.65	0.862	1.51	−5.72 to 8.73	0.689
Severe	5.26	−0.48 to 11.00	0.077	6.12	0.48–11.76	0.040	2.15	−3.30 to 7.59	0.444	2.34	−3.15 to 7.82	0.415
Emotional neglect severity												
Low/moderate	3.40	−1.87 to 8.67	0.212	4.39	−0.82 to 9.59	0.110	1.51	−3.87 to 6.90	0.585	1.06	−4.37 to 6.49	0.708
Severe	3.14	−3.78 to 10.06	0.379	2.76	−3.99 to 9.52	0.435	3.33	−3.26 to 9.92	0.327	3.94	−2.86 to 10.72	0.268
Physical neglect severity												
Low/moderate	−0.55	−6.03 to 4.92	0.845	0.31	−5.06 to 5.68	0.913	7.33	0.77–13.88	0.032	8.10	1.48–14.71	0.021
Severe	6.95	0.92 to 12.99	0.027	6.74	0.86–12.61	0.031	8.03	1.69–14.36	0.015	8.25	1.92–14.58	0.014
*Cultural factors*												
Can speak traditional language												
A little bit	−0.98	−4.03 to 2.08	0.531	−1.06	−4.13 to 2.03	0.502	0.97	−2.20 to 4.13	0.552	0.83	−2.37 to 4.02	0.615
Yes	−5.14	−10.64 to 0.36	0.069	−5.35	−10.79 to 0.07	0.057	2.73	−2.79 to 8.25	0.334	2.62	−2.89 to 8.16	0.356
Family always/often lived by traditional culture	1.52	−1.69 to 4.66	0.339	1.17	−2.14 to 4.27	0.466	0.43	−3.06 to 3.91	0.808	0.53	−3.09 to 4.09	0.773
Traditional language was often/always spoken at home	−2.28	−5.64 to 1.07	0.184	−2.36	−5.72 to 0.96	0.169	1.94	−1.33 to 5.19	0.242	1.42	−1.93 to 4.69	0.403
Often/always lived by traditional culture in past 6 months	−2.69	−5.95 to 0.53	0.102	−2.89	−6.19 to 0.28	0.079	−0.96	−4.47 to 2.54	0.591	−0.96	−4.51 to 2.57	0.597
Participated in traditional ceremonies in past 6 months`	1.08	−1.18 to 3.34	0.350	0.93	−1.33 to 3.21	0.425	2.34	−0.22 to 4.99	0.073	2.51	−0.13 to 5.13	0.064
*Other protective factors*												
Accessed drug/alcohol treatment	2.21	−0.35 to 4.82	0.092	2.57	0.04–5.26	0.051	−2.27	−5.10 to 0.63	0.113	−2.51	−5.33 to 0.50	0.085
Accessed any counseling	2.47	−0.45 to 5.44	0.098	2.77	−0.11 to 5.80	0.065	2.12	−0.83 to 5.11	0.158	2.13	−0.80 to 5.19	0.159
Tried quitting drugs	0.06	−3.53 to 3.62	0.974	0.48	−3.15 to 4.06	0.795	−3.76	−7.24 to −0.26	0.036	−4.33	−7.89 to −0.76	0.018
*Risk factors*												
Slept on streets for >3 nights	−0.11	−3.46 to 3.29	0.950	−0.34	−3.66 to 3.06	0.841	4.43	0.51–8.42	0.026	4.38	0.54–8.42	0.028
Smoking crack daily or more	1.86	−1.66 to 5.40	0.303	1.97	−1.53 to 5.51	0.276	0.18	−3.14 to 3.49	0.917	0.23	−3.16 to 3.61	0.897
Used any drugs in past 6 months	5.31	1.00–9.64	0.017	5.02	0.72–9.34	0.024	5.72	1.59–9.88	0.007	5.88	−1.78 to 10.08	0.006
Injected in past 6 months	2.07	−1.61 to 5.78	0.268	1.62	−2.09 to 5.43	0.399	2.34	−1.19 to 5.87	0.195	2.42	−1.19 to 6.06	0.194
Blackouts from drinking	3.56	0.25–6.89	0.037	3.32	0.01–6.64	0.052	4.67	0.88–8.51	0.017	4.61	0.85–8.50	0.019
Binge drinking	1.25	−2.68 to 5.29	0.530	0.90	−2.96 to 4.98	0.653	1.77	−2.08 to 5.61	0.368	1.84	−2.05 to 5.71	0.356
Sex work involvement	14.42	5.97–22.87	0.001	14.13	5.72–22.56	0.001	5.32	2.17–8.52	0.001	5.19	2.08–8.41	0.001
Inconsistent condom use	−0.09	−3.49 to 3.26	0.958	−0.19	−3.59 to 3.10	0.911	0.12	−3.54 to 3.79	0.950	0.20	−3.46 to 3.90	0.918
Sexually transmitted infection							4.41	−0.80 to 9.63	0.098	3.17	−2.17 to 8.55	0.249
Sexual assault							7.86	0.41–15.39	0.039	7.82	0.49–15.39	0.040
HIV-positive serostatus	−0.17	−6.54 to 6.21	0.958	−0.53	−7.01 to 5.93	0.874	−2.90	−8.71 to 2.91	0.331	−1.49	−7.68 to 4.70	0.644
HCV-positive serostatus	−0.83	−6.37 to 4.70	0.769	0.26	−5.44 to 5.97	0.931	−1.10	−5.87 to 3.67	0.654	−0.06	−5.28 to 5.15	0.983

a Adjusted for age, location, and education.

b Calculated using profile likelihood method.

Several additional risk factors were associated with increased distress among men. Having recently used any drugs was associated with a 1.67-fold increase in distress (*β* = 5.02; percentile shift: 30th to 50th) and reporting recent blackouts from drinking was associated with a 1.4-fold increase in distress (*β* = 3.32; percentile shift: 30th to 42nd). In addition, men who reported recently being involved in sex work experienced 2.7-fold higher distress scores (*β* = 14.13; percentile shift: 30th to 81st).

Notable marginal associations were observed between cultural and other protective factors and psychological distress among men. Being able to speak a traditional language was marginally associated with a 0.53-fold decrease in distress (*β* = −5.35; percentile shift: 30th to 16th). In addition, men who reported recently often/always living by traditional culture had somewhat lower average psychological distress scores (*β* = −2.89; percentile shift: 30th to 20th). Men who had recently accessed drug/alcohol treatment (*β* = 2.57; percentile shift: 30th to 40th) or counseling (*β* = 2.77; percentile shift: 30th to 41st) demonstrated higher mean psychological distress scores. These corresponded to 1.33- and 1.36-fold increases in distress, respectively.

### Factors associated with psychological distress among women

Strong relationships were observed between childhood maltreatment factors and psychological distress among women. Severe emotional abuse was associated with a 1.62-fold increase in distress (*β* = 10.78; percentile shift: 48th to 78th) and severe physical abuse was associated with a 1.37-fold increase in distress (*β* = 5.88; percentile shift: 48th to 66th). In addition, women who had experienced low/moderate (*β* = 8.1) or severe (*β* = 8.25) physical neglect experienced distress scores that were 1.52 higher (percentile shift: 48th to 73rd).

For cultural and other protective factors, a marginally significant association was found between recently attending traditional ceremonies and elevated distress levels, translating to a onefold increase in distress (*β* = 2.51; percentile shift: 48th to 50th). A stronger association was observed between recently trying to quit using drugs which corresponded to a 0.57-fold decrease in distress (*β* = −4.33; percentile shift: 48th to 27th).

Several risk factors were associated with elevated psychological distress scores among women. Recent homelessness was associated with a 1.1-fold increase in distress (*β* = 4.38; percentile shift: 48th to 52nd). Having recently used any drugs was associated with a 1.4-fold increase in distress (*β* = 5.88; percentile shift: 48th to 67th) and recent blackouts from drinking were associated with a 1.2-fold increase in distress (*β* =  4.61; percentile shift: 48th to the 57th). Recent sex work involvement correlated with a 1.4-fold increase in distress (*β* = 5.19; percentile shift: 48th to 66th). Finally, women who reported recently having been sexually assaulted experienced a 1.55-fold increase in distress (*β* = 7.82; percentile shift: 48th to 73rd).

### Combined multivariable model

Combined multivariable LME models assessed which factors had the greatest impact on psychological distress (Tables 5 and 6). Among men, factors that remained significant at the *p* < 0.05 level included low/moderate (*β* = 9.05) and severe (*β* = 8.29) childhood emotional abuse. These factors represented a 2.2-fold increase in distress (percentile shift: 30th to 66th). In addition, men who reported having had blackouts from drinking had a 1.63-fold increase in distress (*β* = 3.79; percentile shift 30th to 46th). In this model, 21% of the variance was explained by the fixed effects and 73% of the variance was explained by both fixed and random effects. Among women, factors that remained significant at the *p* < 0.05 level included having experienced severe childhood emotional abuse which increased mean distress scores by 1.3-fold (*β* = 13.44; percentile shift: 48th to 61st). Women who had recently used any drugs (*β* = 5.64) or had blacked out from drinking (*β* = 4.67) on average had a 1.31-fold increase in distress (percentile shift: 48th to 63rd). In this model, 25% of the variance was explained by the fixed effects and 69% of the variance was explained by both fixed and random effects.

**Table 5.** Multivariate final model for males.

	*β*	95% CI	*p*
Mean age (s.d)	−0.50	−0.99 to 0.01	0.054
Location (Vancouver)	5.56	0.17–10.95	0.055
High school education or higher	4.60	−1.92 to 11.12	0.186
Emotional abuse severity			
Low/moderate	9.05	3.46–14.65	0.003
Severe	8.29	1.83–14.74	0.018
Blackouts from drinking	3.79	0.35–7.13	0.032

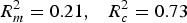
.

**Table 6.** Multivariate final model for females.

	*β*	95% CI	*p*
Mean age (s.d)	−0.20	−0.73 to 0.32	0.463
Location (Vancouver)	−0.34	−6.00 to 5.30	0.910
High school education or higher	−1.99	−10.36 to 6.37	0.654
Emotional abuse severity			
Low/moderate	2.94	−4.12 to 9.99	0.434
Severe	13.44	6.73–20.11	<0.001
Used any drugs in past 6 months	5.64	0.79–10.49	0.028
Blackouts from drinking	4.67	0.92–8.60	0.020

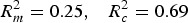
.

## Discussion

This study explored longitudinal risk and protective factors related to psychological distress among young Indigenous people who have been impacted by substance use in three cities in BC, Canada. We observed psychological distress levels among participants that were comparable to a norm sample of adult psychiatric outpatients. This was especially true for the young Indigenous women in the study. In addition, we found concerning relationships between childhood maltreatment and substance use patterns with elevated psychological distress, which corresponded to substantial shifts toward higher percentile rankings of distress within the psychiatric outpatient sample. Nevertheless, important protective factors were associated with decreased distress and warrant consideration.

### Comparing mean psychological distress with other studies

Comparing psychological distress scores with other samples is difficult, as mental health is dynamic and influenced by many intersecting factors. In addition, few studies have used the SCL-90-R within Indigenous populations. Our participant enrollment process was not based on mental health, so it is not surprising that the raw average psychological distress scores observed in this study were lower compared with averages reported in studies involving 273 American Indian adults who had been clinically diagnosed with depression or anxiety (Davis *et al*., 2011) and involving 54 American Indian youth who had attempted suicide (Howard-Pitney *et al*., 1992). Consistent with other research, we observed gender differences in distress, with women scoring significantly higher than men for each symptom dimension (Gold *et al*., 1999).

### Risk factors and psychological distress

Concerning associations were found between childhood emotional abuse, physical abuse, sexual abuse, and physical neglect with substantial elevations in psychological distress. These findings align with population-based research demonstrating the devastating effect of these specific types of maltreatment on social/personal distress, and stress-coping with substances over the life course. Childhood emotional abuse has been shown to be associated with psychiatric diagnoses, lower self-esteem, unhappy intimate relationships, and illicit drug use (Mullen *et al.*, 1996; Dube *et al.*, 2003; Edwards *et al.*, 2003). Childhood physical abuse has a profoundly negative association with depression, anxiety, and anger (Springer *et al*., 2007), while childhood physical neglect is associated with depression and drug use (Schilling *et al*., 2007). Childhood sexual abuse has been strongly associated with depression, anti-social behavior, and illicit drug use among young adults in a longitudinal population study (Schilling *et al*., 2007). These results are also consistent with previous Cedar Project research that linked childhood maltreatment with high-risk outcomes including binge injection drug use, inconsistent condom use, and HCV infection (Pearce, 2014). Indigenous Elders and leaders in Canada, the USA, and Australia have emphasized the importance of acknowledging that Indigenous family violence is an artifact of the residential/boarding school and child welfare systems and have called on mental health interventions to address colonization as a determinant of ongoing stress (Strickland *et al*., 2006; Hocking, 2008; Christian, 2010; Reading, 2015). Mental wellness programs must therefore employ culturally safe and healing-centred best practices that engage Indigenous young people who use drugs into healthier stress-coping strategies. In addition, such programs must meaningfully address concurrent systemic factors that contribute to distress such as experiences of racism, discrimination within healthcare, and interpersonal violence (Evans-Campbell *et al.*, 2006; Harris *et al.*, 2006; Currie *et al.*, 2013*b*).

Recently experiencing homelessness or sexual assault was associated with markedly higher levels of psychological distress among young Indigenous women in the current study. These findings are consistent with previous research involving ethnic minority women. For example, in a cohort of ethnically diverse homeless women in the USA, Tsai *et al*. (2015) demonstrated that those who had experienced recent violent victimization were more likely to experience poorer mental health and psychiatric hospitalization. In a previous Cedar Project study, sexual assault was the strongest predictor of lower mean resilience scores among participants (Pearce *et al*., 2015*b*). National surveys from Canada, the USA, and Australia indicate that Indigenous women experience dramatically higher rates of sexual assault than non-Indigenous women (Statistics, 2006; Bachman *et al*., 2010; Brennan, 2011) and most do not seek help from police or mental health services (McCalman *et al*., 2014; Pearce *et al*., 2015*a*). In all countries, meaningful collaboration between Indigenous leadership, government/non-governmental bodies, and public health is urgently required to develop culturally safe sexual assault prevention and intervention strategies that support Indigenous women's strength and resilience.

Sex work was associated with substantial increases in psychological distress among men and moderate increases among women. Few studies have addressed mental health of people involved in sex work (Rossler *et al*., 2010; Surratt *et al*., 2012); yet, Indigenous women are over-represented in street-based sex work in BC (Mehrabadi *et al*., 2008; Deering *et al*., 2014). Lack of safety due to violence, stigmatization, and criminalization of people involved in sex work may contribute to psychological distress (Krusi *et al*., 2010). In light of these findings, qualitative inquiry into the mental health needs of Indigenous people involved in sex work that focuses on facilitators of mental wellness is warranted.

Finally, recent drug use and experiencing blackouts while drinking were each associated with moderately increased psychological distress for both men and women. Moreover, in the parsimonious models, we determined that childhood emotional abuse and recent blackouts from drinking were associated with elevated distress among men, and that emotional abuse, recent drug use, and recent blackouts from drinking were associated with elevated distress among women. Establishing the temporal sequence between substance use and mental health is problematic, but studies have suggested that poor mental health precedes substance use (rather than *vice versa*) (Bell & Britton, 2014). Young Indigenous people may therefore be using substances to self-medicate the complex array of challenges to their mental wellness, a theory supported by Cooper's Motivational Model of substance use (Cooper *et al*., 1995). Moreover, Walters and Simoni's (2002) ‘Indigenist stress-coping paradigm’ conceptualizes the cumulative impact of historical, social, and economic stressors on Native American women, related psychiatric and chronic/infectious disease outcomes, and the potential preventative or mitigating role of ‘cultural buffers’ that offer positive stress-coping alternatives to substance use. It is highly encouraging that interventions in the USA, Canada, and New Zealand are reporting that culturally based mental health programming improves trauma, addictions, and mental health outcomes among Indigenous peoples (Goodkind *et al*., 2012; Mathieson *et al*., 2012; Marsh *et al*., 2015).

### Strengths-based factors and psychological distress

It is important to note that we observed marginally significant (*p* < 0.10) relationships between being able to speak a traditional language and having recently lived by traditional culture with lower mean psychological distress scores among young men in this study.

Lack of significance in these results may reflect small sample size or that Cedar Project participants continue to experience cultural disconnections as a result of intergenerational impacts of residential school and child welfare experiences, and therefore fewer participants reported lifetime or recent cultural engagement. Larger studies are needed to examine these possible associations. Nevertheless, these findings are consistent with previous Cedar Project research which demonstrated that participants who knew how to speak their traditional language and often lived by traditional culture had higher mean resilience scores (Pearce *et al*., 2015*b*). Further, these findings support qualitative studies from New Zealand involving Indigenous (Māori and Samoan) peoples which highlight the critical importance of language retention and connection to culture to mental wellbeing (Tamasese *et al*., 2005; Puna & Tiatia-Seath, 2017). It is also notable that we found marginal associations between women's recent participation in traditional ceremonies and men's recent access to drug/alcohol treatment as well as counseling with somewhat elevated distress scores. It is possible that participants were connecting with these services and cultural practices in response to their stress, and were seeking help to support their mental health and wellness. It is also important to consider literature from Canada and Australia highlighting that Indigenous people who identify strongly with their Indigenous culture experience greater happiness and lower illicit drug use, yet also more racial discrimination and subsequent distress (Dockery, 2011; Currie *et al*., 2013*a*). For example, Currie *et al*. (2012) demonstrated that among 60 Indigenous university students in Canada, two-thirds had experienced high levels of racial discrimination, and that those who identified as being traditional or cultural Indigenous persons were significantly more likely to experience racial discrimination. Conversely, women in the Cedar Project who had recently tried to quit using drugs experienced lower psychological distress. Extant literature from Australia, New Zealand, and Canada has highlighted the deficiencies of mental health programs and professionals that serve Indigenous peoples including failure to: develop the prerequisite skills, knowledge, and attitudes for culturally competent/safe care; create family-centered care models that feel welcoming/non-judgmental; offer long-term, holistic care plans; and embrace principles of harm reduction for those with comorbid problematic substance use (Papps & Ramsden, 1996; Wardman & Quantz, 2006; Sones *et al*., 2010; Westerman, 2010; Pearce, 2014). Globally, Indigenous leaders in mental health research and practice have called for balance between western and Indigenous approaches to mental health practice (Sones *et al*., 2010). This has the potential to facilitate healing from intergenerational trauma as well as support positive stress-coping and resistance against structural and interpersonal violence. For young Indigenous people who use drugs in BC, expanded and sustainable support is needed for balanced drug and alcohol treatment programs that address social determinants, utilize evidence-based clinical interventions, and embrace traditional Indigenous therapies for mental wellness (First Nations Health Authority, 2013).

### Limitations

There are important limitations to this study. Data were self-reported and obtained from a non-probabilistic street-involved sample. Selection bias may have been introduced if, for example, there were young Indigenous people who were not connected to services in the downtown areas of study locations and therefore unreachable to the study team. We cannot rule out selection bias and its impact, but are confident that our recruitment methods and rigorous eligibility criteria ensured that our sample is representative of Indigenous young people who use drugs in Vancouver, Prince George, and Chase. There is potential for recall bias, socially desirable reporting, and misclassification (except for HIV and HCV). For example, childhood maltreatment and sex work may have been under-reported. Responses to historical questions may be influenced by the participants’ ability to recall event(s) and the effect of memory on these study variables is difficult to assess. Despite these limitations, we believe that this study provides newly reported and important epidemiological evidence about risk and strengths-based factors associated with psychological distress among young Indigenous people who use drugs.

## Conclusion

Psychological distress among young Indigenous people who use drugs is a deeply concerning public health issue that must be understood within the context of colonization as a social determinant of health. Lasting funding is needed for community-driven mental wellness interventions that address the root causes of interpersonal and systemic violence and offer opportunities to access Indigenous culture, traditions, languages, medicines, and spirituality (Clark, 2016). Such responses to mental wellness must be rooted in Indigenous self-determination for the health and wellbeing of Nations, communities, and families.
